# Ultrasonication-mediated fabrication of artificial oleosomes from hemp seed oil-body proteins and rose essential oil for banana preservation

**DOI:** 10.1016/j.ultsonch.2025.107407

**Published:** 2025-05-28

**Authors:** Haoran Zhu, Yuhan Cao, Xinyu Zhang, Qin Hu, Feng Xue

**Affiliations:** a School of Pharmacy, Nanjing University of Chinese Medicine, 138 Xianlin Road, Nanjing 210023, PR China; b Jiangsu Key Laboratory of Medicinal Substance and Utilization of Fresh Chinese Medicine, Nanjing University of Chinese Medicine, 138 Xianlin Road, Nanjing 210023, PR China

**Keywords:** Hemp seed oil body proteins, Artificial oleosomes, Ultrasonication, Essential oil, Coatings, Preservation

## Abstract

This study systematically evaluates the efficacy of three emulsification strategies (ultrasonication, high-pressure homogenization, and high-speed shearing) in fabricating artificial oleosomes (AOs) stabilized by hemp seed oil body proteins (OBPs) and rose essential oil. Comparative analyses revealed that ultrasonication generated AOs with superior physicochemical attributes, including reduction in mean particle size, higher ζ-potential, and increased surface hydrophobicity compared to conventional methods. These structural enhancements correlated with improved functional performance: ultrasonicated AOs exhibited lower apparent viscosity, higher radical scavenging capacity, and reduction in light transmittance. Coatings derived from ultrasonicated AOs demonstrated exceptional barrier properties, achieving better water vapor permeability inhibition and oxygen transmission reduction relative to other coatings. Practical validation on bananas revealed that ultrasonicated AOs coatings extended shelf life through enzymatic browning suppression, moisture retention and content of soluble solids maintaining. The findings establish ultrasonication as a scalable strategy for engineering multifunctional AOs with applications in sustainable food preservation.

## Graphical abstract

**Figure:**
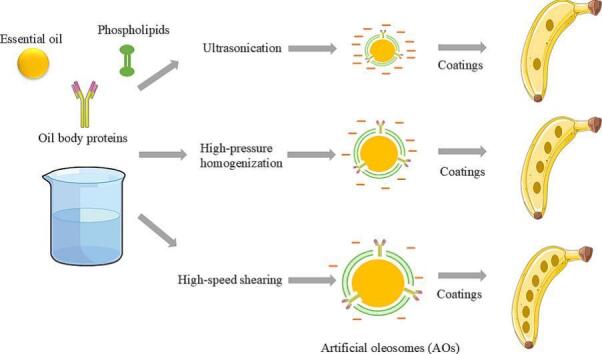

## 1 Introduction

Hemp (Cannabis sativa L.), originating in Asia, represents one of humanity’s oldest cultivated crops, with archaeological evidence tracing its use in China back to 5000–6000 BCE [1]. Traditional Chinese medicine has long employed hemp seed to treat various conditions, including hypertension [2] and constipation [3], while modern research has revealed their value as sources of bioactive peptides [4] and functional food components [5]. Recent scientific investigations have extensively characterized hemp seed protein isolate (HPI), elucidating its functional properties including solubility, emulsification capacity, foaming characteristics, gelation behavior, and film-forming ability [[6], [7], [8], [9]]. However, the oil body proteins (OBPs) from hemp seed remain significantly understudied despite their technological potential.

OBPs comprise three amphipathic structural proteins (oleosin, caleosin, and steroleosin) that anchor within the phospholipid monolayer of oleosomes [10]. These proteins confer stability to natural oleosomes through combined steric hindrance and electrostatic repulsion mechanisms [11]. Their remarkable stabilization capacity in plant seed suggests potential utility in engineered oil-in-water emulsion systems [12]. Consequently, OBPs-stabilized emulsions have emerged as a research focus, particularly for artificial oleosome (AOs) fabrication. AOs have sustained decades of investigation due to their exceptional promise for food and pharmaceutical applications. As delivery vehicles for bioactive compounds, AOs demonstrate superior physicochemical stability, excellent biocompatibility, and enhanced bioavailability [13]. In biotechnological applications, AOs serve as versatile platforms for protein purification, refolding, and immobilization, offering process simplification and yield improvement over conventional methods [13].

Essential oils (EOs) − volatile secondary metabolites extracted from plant materials (leaves, flowers, roots) − exhibit complex chemical profiles with demonstrated antioxidant and antimicrobial activities [14]‌. These properties have motivated their investigation as natural preservatives in food systems, with oregano, grapefruit, lemon, rose, camellia, and mustard oils showing particular promise [[15], [16], [17]]. In food preservation systems, EOs are conventionally employed either as emulsified formulations [18] or through integration into polymer-based matrices [19]. The remarkable emulsification capacity and structural stability of natural oleosomes prompted our investigation into AOs as novel delivery vehicles for EO encapsulation in food preservation applications. Despite their demonstrated potential in other delivery systems, the specific application of AOs for EOs encapsulation in food preservation contexts remains substantially underexplored in the current literature.

Ultrasonication has become the predominant AOs preparation method, where cavitation effects promote formation of stable OBPs-phospholipid adsorption layers around triacylglycerol cores [13]. This process simultaneously induces protein partial unfolding, enhancing interfacial stability, while mechanical effects reduce particle size for improved system stability. Alternative emulsification techniques including high-pressure homogenization and high-speed shearing offer different processing advantages [20], yet comparative studies of their effects on AO properties are notably absent from the literature.

This investigation therefore examines hemp seed OBPs as structural components for essential oil-loaded AOs, with three key objectives: characterize how emulsification method (ultrasonication vs. high-pressure homogenization vs. high-speed shearing) influences AOs physicochemical properties (particle size distribution, ζ-potential, surface hydrophobicity, rheological behavior); evaluate the resulting AOs’ performance as edible coatings through comprehensive microstructural and functional analysis; assess practical efficacy in extending fruit shelf-life as a model food preservation application.

## 2 Materials and methods

### 2.1 Materials

The hemp seed (*Cannabis sativa* L.) sample was purchased from Yunnan Industrial Hemp Co., Ltd. (Kunming, Yunnan, China). Essential oil from rose petals (95 %), phospholipids (from soybean, 98 %), 1,1-Diphenyl-2-picrylhydrazyl (DPPH) and 2,2′-Azinobis-(3-ethylbenzthiazoline)-6-sulfonic acid (ABTS) radicals were purchased from Shanghai Yuanye biotechnology Co., Ltd. (Shanghai, China). The bananas were purchased from a local supermarket (Nanjing, China). The other reagents used were purchased from Sinopharm Chemical Reagent Co., Ltd. (Shanghai, China).

### 2.2 Extraction of oil body proteins (OBPs) from hemp seed

Oil bodies were extracted from hemp seed following previously established protocol with minor modifications [21]. Briefly, the hemp seed were soaked in a sodium bicarbonate solution overnight, followed by homogenization using a homogenizer. The homogenate was then centrifuged at 10,000 rpm and 4°C for 30 min, and the upper oil body layer was collected for subsequent protein extraction. The oil bodies were mixed with a petroleum ether-ethanol solution and centrifuged again at 10,000 rpm and 4°C for 30 min. The intermediate layer, containing the oil body proteins, was collected and freeze-dried (LABCONCO, Kansas City, MO, USA) to obtain hemp seed OBPs.

### 2.3 Molecular weight of hemp seed OBPs

The analysis of hemp seed OBPs profiles was performed according to previously described methods with minor modifications [21]. Hemp OBPs were dissolved in loading buffer at a concentration of 4 mg/mL. Protein composition was analyzed using SDS-PAGE electrophoresis (Bio-Rad Laboratories Inc., Hercules, USA) with 12 % separating gel. Samples (10 μL) were loaded and electrophoresed at 200 V for 30 min. Protein bands were visualized by Coomassie Brilliant Blue R250 (Servicebio, Wuhan, China), followed by destaining to observe protein patterns.

### 2.4 Preparation of artificial oleosomes (AOs)

AOs were synthesized through modified protocols adapted from previous study [21], comprising three distinct emulsification strategies. Initially, the 1.5 g OBPs and 1 g phospholipids were homogenized in 87.5 mL deionized water (1000 rpm, 30 min) using a magnetic stirrer (Shanghai Yidian Scientific Instrument Co., Ltd, China), during which 10 g rose essential oil was incrementally emulsified. The colloidal systems were subsequently processed by: (A) High-speed homogenization (IKA Instrument Co., Ltd., Guangzhou, Guangdong, China) at 15,000 rpm for 2 min; (B) High-pressure homogenization (Ningbo Xinzhi Biotechnology Co., Ltd., Ningbo, China) at 100 MPa for 2 cycles; (C) Ultrasonic treatment (NingBo Scientz Biotechnology Co. Ltd., China) using a titanium probe (Φ 6.35 mm) at 20 kHz/400 W with 2 s pulse intervals for 20 min. All procedures were thermally regulated using an ice-water bath. The resultant emulsions were designated as shear-derived (AOs-S), pressure-derived (AOs-P), and ultrasonicated (AOs-U) AOs, respectively.

### 2.5 Characterization of AOs

The AOs were diluted with distilled water to a final concentration of 0.1 % (v/v) to minimize multiple scattering effects. Particle size was determined using a laser diffraction particle size analyzer (Beckman Coulter Inc., LS 13,320, USA), with refractive indices set at 1.33 for the dispersant (water) and 1.50 for the particles. The ζ-potential was measured using a Malvern Mastersizer 3000 instrument (Malvern Instruments Ltd, Worcestershire, UK). For surface hydrophobicity analysis, AOs were diluted with phosphate-buffered saline (PBS, 0.1 M, pH 7.0) to concentrations ranging from 0.5 to 10 μ L/mL. Subsequently, 4 mL of each diluted solution was mixed with 50 µL of 1-anilino-8-naphthalenesulfonate (8 mM) and incubated in darkness for 15 min. Fluorescence intensity was measured using excitation and emission wavelengths of 390 nm and 470 nm, respectively. Surface hydrophobicity was quantified by plotting fluorescence intensity against AOs concentration and calculating the slope of the resulting linear regression curve [22]. The rheological properties of AOs were characterized using a rotational rheometer (HR-1, TA Instruments, Leatherhead, UK) equipped with a parallel plate geometry (diameter: 60 mm; gap: 1000 μm). To evaluate the flow behavior, a continuous shear test was performed by systematically varying the shear rate from 0.1 to 1000 s^−1^, allowing for the determination of viscosity as a function of shear rate. All measurements were conducted under controlled temperature conditions (25 °C) to ensure data reproducibility and accuracy. For microscopic observation, AOs were subjected to a 5-fold dilution to ensure optimal visualization. Subsequently, the samples were stained using fluorescein isothiocyanate (FITC) at a concentration of 0.1 mg/mL, dissolved in dimethyl sulfoxide (DMSO), to label the protein components. Concurrently, Nile red, also at a concentration of 0.1 mg/mL and dissolved in DMSO, was employed to specifically stain the lipid constituents. A 20 µL aliquot of the stained emulsion was placed on a microscope slide, covered with a coverslip, and examined using an inverted fluorescence microscope (DMC5400, Leica Microsystems, Germany) equipped with a 40 × objective lens [23]. The free radical scavenging activity was assessed according to a previously established method [24]. Briefly, AOs were diluted with ethanol or (NH_4_)_2_S_2_O_8_ (2.45 mM) solution at a ratio of 1:50 (v/v). Two milliliters of the resulting sample were mixed with 2 mL of DPPH (0.2 mM) or ABTS solution (7 mM). The mixtures were incubated in the dark for 30 min at 37 °C, and absorbance was measured at 517 nm (for DPPH) or 734 nm (for ABTS) using a spectrophotometer (Spark 10 M, Tecan, Switzerland).

### 2.6 Characterization of AOs coatings

AOs coatings were fabricated by immersing transparent glass slides, gas-permeable membranes (cellulose acetate), polytetrafluoroethylene (PTFE) plates, or silicon substrates in AOs for 30 s, followed by air-drying at room temperature for 15 min. The optical transparency was evaluated by measuring the light transmittance through AOs-coated glass slides in the wavelength range of 200 to 1000 nm using a UV–Vis spectrophotometer (Spark 10 M, Tecan, Switzerland). Water vapor permeability (WVP) was determined by monitoring the weight change of silica gel in a permeation cup sealed with AOs-coated gas-permeable membranes [15]. The oxygen barrier property was assessed by measuring the peroxide value (PV) of oil stored in permeation cups sealed with AOs-coated gas-permeable membranes [25]. Surface wettability was characterized by measuring the contact angle of deionized water (5 μL) droplets on AOs-coated PTFE plates using goniometer (OCA15EC, Stuttgart, Germany). Droplet images were captured and analyzed using Image J software to calculate the contact angle. For analysis of intermolecular interactions, AOs coatings were peeled off from PTFE plates and scanned using Fourier transform infrared spectroscopy (FTIR-7600, Lambda, Australia) in the spectral range of 500–4000 cm^−1^ with a resolution of 4 cm^−1^. Delaminated AO coatings from PTFE plates were also analyzed for their thermal properties. Thermal property was evaluated by differential scanning calorimetry (DSC) using a thermal analyzer (TA Instruments, USA) under a nitrogen atmosphere, with temperature ramping from 25 to 250 °C at a rate of 10 °C/min. The surface morphology of AOs-coated silicon substrates was examined using a Regulus 8100 scanning electron microscope (HITACHI, Japan) operated at an acceleration voltage of 5.0 kV. Additionally, surface topography was analyzed using a Bruker Dimension Icon atomic force microscope (Karlsruhe, Germany) to obtain high-resolution nanoscale images. The data were processed with Nanoscope Analysis (Version 1.5, Bruker) to calculate root mean square roughness (Rq) [26].

### 2.7 Evaluation of the preservation effect of AOs coatings on bananas

To evaluate the preservation efficacy of AOs, bananas were selected as the model fruit. The bananas were initially immersed in AOs for 30 s to ensure uniform coating application, followed by air-drying at room temperature for approximately 15 min [27]. The preservation and quality-enhancing effects of AOs on cherry bananas were subsequently assessed under controlled conditions. The samples were maintained under ambient temperature conditions (25 ± 1 °C), and their visual appearance, whiteness, weight loss and total soluble solids were evaluated after a 5-day storage period. Colorimetric analysis was performed using a Hunter-Lab colorimeter (Reston, VA) to measure the key color parameters, and the whiteness index was calculated based on a previously established methodology [15]. Total soluble solids were measured by refractometer (Guangzhou Suwei Electronic Technology Co., Ltd, China).

### 2.8 Statistical analysis

All experiments were conducted in triplicate, and the results are presented as mean ± standard deviation (SD). Statistical analysis was performed using one-way analysis of variance (ANOVA) implemented in SPSS software (version 25.0, SPSS Inc., Chicago, IL, USA). To determine significant differences among groups, Tukey’s test was used to establish the significance differences among the mean values at 0.95 confidence level.

## 3 Results and discussion

### 3.1 Molecular weight of OBPs from hemp seed

**Fig. 1:**
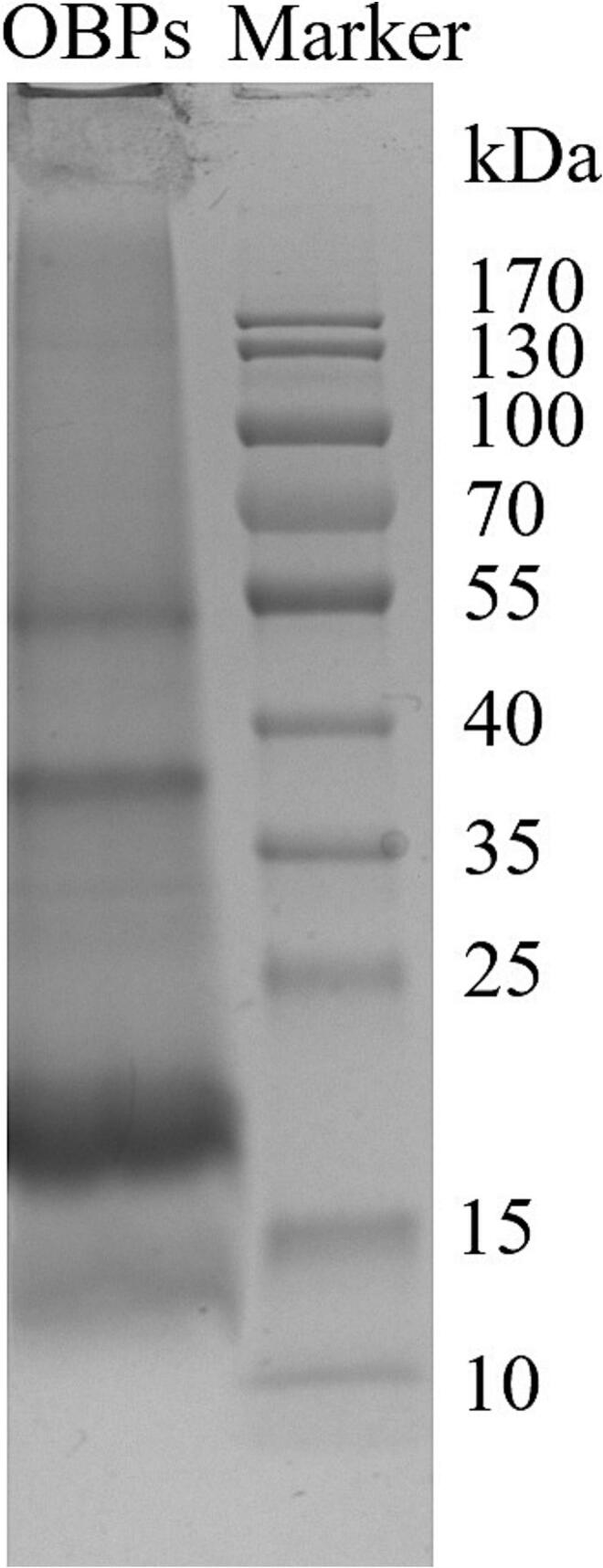
Sodium dodecyl sulfate–polyacrylamide gel electrophoresis of hemp seed oil body proteins (OBPs).

The OBPs are typically composed of three major components: oleosins, caleosins, and steroleosins, with molecular weights ranging from 15 to 26 kDa, 27–35 kDa, and greater than 35 kDa, respectively [28]. Among these proteins, oleosins are considered the primary contributors to the emulsifying properties of OBPs due to their excellent amphiphilic characteristics [29]. As illustrated in Fig. 1, protein profile of hemp OBPs is primarily distributed within the range of 15–25 kDa, indicating that oleosins constitute the major component of hemp seed OBPs. This observation is consistent with previous reports on oleosins from soybean and rapeseed OBPs, where soybean oleosins were identified within the range of 16–24 kDa [30], rapeseed oleosins within the range of 17–20 kDa [31]. However, significant variations in molecular weight have been observed among oleosins derived from different plant sources. For instance, oleosins from pumpkin seeds exhibit a molecular weight of 14.5 kDa [32], while those extracted from cucumber seeds also include isoforms with molecular weights below 15 kDa [21]. Notably, oleosins from those sources have demonstrated superior emulsifying properties and have been extensively utilized in the preparation of emulsions [33] or as carriers for hydrophobic compounds [34]. Furthermore, protein bands with apparent molecular weights of approximately 37 kDa and 54 kDa were tentatively identified as caleosin and steroleosin, respectively. This observation is consistent with previous reports characterizing the OBPs in hemp seed [35].

### 3.2 Particle size of AOs

**Fig. 2:**
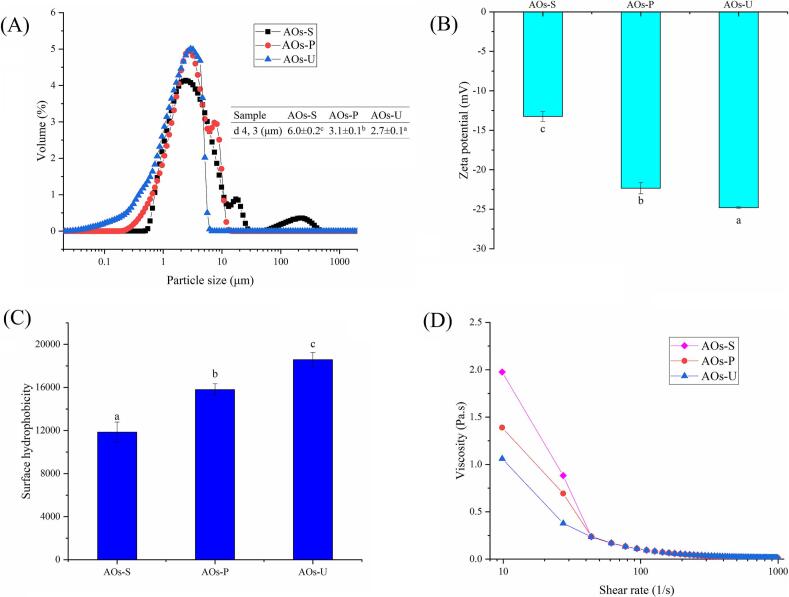
Particle size (A), ζ-potentials (B), surface hydrophobicity (C) and apparent viscosity (D) of artificial oleosomes (AOs) stabilized by hemp seed oil body proteins (OBPs). AOs-S: AOs fabricated by high-speed shearing; AOs-P: AOs fabricated by high-pressure homogenization; AOs-U: AOs fabricated by ultrasonication. Results with different letters within the same pattern are significantly different (p < 0.05).

The physical stability of emulsions is fundamentally governed by their particle size distribution, with smaller particle sizes being indicative of enhanced stability [36]. As demonstrated in Fig. 2 A, a comparative analysis of three emulsification techniques (ultrasonication, high-pressure homogenization, and high-speed shearing) revealed significant differences in the particle sizes of the resulting AOs. Specifically, ultrasonication yielded AOs with markedly smaller particle sizes compared to those produced by high-pressure homogenization, while high-pressure homogenization generated particles significantly smaller than those obtained through high-speed shearing. This phenomenon can be attributed to the mechanism of acoustic cavitation, a physical process intrinsic to ultrasonic emulsification [37]. Acoustic cavitation involves the formation, growth, and implosive collapse of microbubbles within the emulsification system, which intensifies intermolecular interactions and facilitates the reduction of particle size, thereby producing emulsions with enhanced uniformity and physical stability. As shown in Fig. 1 A, the ultrasonically prepared AOs exhibited a monomodal size distribution, whereas the other two types of AOs displayed multimodal distributions. These findings align with previous studies on myofibrillar protein-stabilized emulsions, where ultrasonication and high-pressure homogenization were shown to produce emulsions with significantly smaller particle sizes than high-speed shearing, a result ascribed to the instantaneous high pressure and cavitation effects inherent to ultrasonication [38]. Furthermore, similar observations were reported in the preparation of double-layer emulsions, where ultrasonication consistently produced emulsions with smaller particle sizes compared to high-pressure homogenization [39]. These results collectively underscore the superior efficacy of ultrasonication in achieving emulsions with optimal stability and particle size characteristics. The observed changes in particle size typically indicate corresponding alterations in surface groups. Consequently, we will subsequently investigate the effects of these three methods on both the surface charge density and hydrophobicity of the proteins.

### 3.3 Zeta potential of AOs

The stability of emulsions is fundamentally governed by their surface charge density, with higher net surface charge generally conferring greater stability through enhanced electrostatic repulsion between emulsion droplets, thereby effectively preventing flocculation and coalescence [40]. As illustrated in Fig. 2 B, all AOs exhibited negative surface potentials, indicative of the presence of negatively charged groups on their surfaces. The emulsification method exerted a significant influence on the surface charge density of the AOs, with ultrasonication demonstrating superior efficacy in generating higher surface charge density compared to high-pressure homogenization or high-speed shearing. This phenomenon can be attributed to the cavitation effect of ultrasonication, which prevents the formation of aggregates and avoids the shielding of charged groups. Previous study on protein-based emulsions has similarly demonstrated that droplets with smaller particle sizes exhibit higher surface charge densities [17]. Conversely, high-pressure homogenization yielded lower surface charge density relative to ultrasonication, potentially due to its comparatively limited impact on protein structural modification or the induction of protein aggregation under high pressure, which might cause some charged groups on the protein surface to be buried [41]. Our result is also consistent with earlier investigations on myofibrillar protein-stabilized emulsions, which demonstrated that both ultrasonication and high-pressure homogenization produced emulsions with significantly elevated surface charge density compared to those prepared by high-speed shearing [38]. These collective findings underscore the critical role of emulsification methodology in modulating surface charge properties and, consequently, emulsion stability.

### 3.4 Surface hydrophobicity of AOs

The surface hydrophobicity of emulsions, a critical determinant of their stability, is predominantly governed by the structural configuration of proteins adsorbed at the oil–water interface [42]. As illustrated in Fig. 2 C, AOs fabricated via ultrasonication demonstrated markedly higher surface hydrophobicity relative to those produced by high-pressure homogenization and high-speed shearing. This phenomenon can be ascribed to the exposure of latent hydrophobic moieties within the protein matrix, suggesting that ultrasonication facilitates the structural unfolding of proteins. The revelation of these hydrophobic groups augments the adsorption efficiency of interfacial proteins, thereby fostering the formation of a viscoelastic interfacial film that effectively impedes droplet coalescence [43]. The underlying mechanism involves ultrasonication-induced cavitation, microstreaming, and bubble collapse, which collectively contribute to the exposure of hydrophobic domains on the droplet surface [44]. This observation aligns with prior investigations on myofibrillar protein-stabilized emulsions, wherein ultrasonication was shown to enhance the exposure of hydrophobic amino acids, consequently elevating the surface hydrophobicity of the resultant emulsions [45,46]. Moreover, our experimental data corroborate existing literature, confirming that both ultrasonication and high-pressure homogenization yield emulsions with superior surface hydrophobicity compared to high-speed shearing [38]. Notably, however, this study revealed no statistically significant disparity in surface hydrophobicity between emulsions prepared by ultrasonication and high-pressure homogenization. This parity may be attributed to the precise regulation of energy input in both methodologies, which likely induced comparable modifications to the adsorbed proteins.

### 3.5 Rheological properties of AOs

As depicted in Fig. 2 D, all AOs exhibited shear-thinning behavior, a hallmark of non-Newtonian fluids. This effect arises due to the molecular cross-linking within the emulsion system under static conditions. Upon the application of shear stress, the clustered fat globules become dispersed, resulting in a stabilization of the apparent viscosity as the molecular configuration attains a state of equilibrium [47]. Notably, the AOs prepared through ultrasonication displayed the lowest apparent viscosity, followed by those generated via high-pressure homogenization, whereas those produced by high-speed shearing exhibited the highest apparent viscosity. This observation is consistent with prior research on myofibrillar protein-stabilized emulsions, which demonstrated that ultrasonication consistently results in emulsions with reduced apparent viscosity compared to those prepared by high-pressure homogenization and high-speed shearing [38]. The underlying mechanism for this phenomenon may be attributed to the distinct particle size distributions achieved by the three preparation methods. Specifically, ultrasonication typically yields AOs with smaller particle sizes (as shown in Fig. 2 A), which enhances their fluidity and consequently reduces apparent viscosity. Previous study also showed that emulsion with smaller particle size exhibited lower apparent viscosity [39]. Furthermore, ultrasonication may contribute to the reduction of apparent viscosity by attenuating intermolecular interactions between oil droplets, thereby facilitating smoother flow dynamics [48]. To validate this hypothesis, we conducted microscopic structural analysis of the AOs.

### 3.6 Microstructure of AOs

**Fig. 3:**
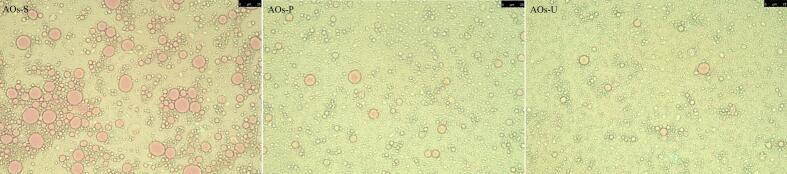
Microstructure of artificial oleosomes (AOs) stabilized by hemp seed oil body proteins (OBPs). AOs-S: AOs fabricated by high-speed shearing; AOs-P: AOs fabricated by high-pressure homogenization; AOs-U: AOs fabricated by ultrasonication.

As depicted in Fig. 3, the pink regions denote essential oil droplets, whereas the light green areas signify OBPs. The preparation of AOs via high-speed shearing predominantly yielded large-sized oil droplets, indicating the method's inefficacy in generating uniform AOs. Conversely, the application of high-pressure homogenization and ultrasonic emulsification resulted in samples primarily composed of small light green particles, a phenomenon particularly evident in ultrasonically prepared AOs. This outcome is attributed to the establishment of a uniform adsorption layer on the AOs' surface, which impedes the interaction of fluorescent dyes with the oil droplets, thus revealing only the color of the surface protein adsorption layer. These findings underscore the efficacy of high-pressure homogenization and ultrasonication in encapsulating oil droplets within the interfacial film formed by OBPs. Such observations corroborate prior research on myofibrillar protein-stabilized emulsions, which noted that high-speed shearing tends to produce larger oil droplets, while high-pressure homogenization and ultrasonication markedly reduce droplet size [38]. Additionally, the microscopic findings are in agreement with the particle size measurement results (as shown in Fig. 2 A), reinforcing the validity of the observed phenomena.

### 3.7 Antioxidant activity of AOs

**Fig. 4:**
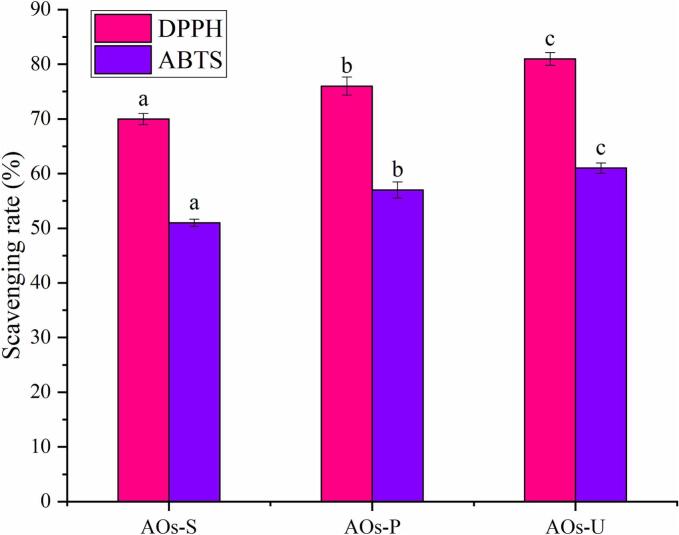
Antioxidant activities of artificial oleosomes (AOs) stabilized by hemp seed oil body proteins (OBPs). AOs-S: AOs fabricated by high-speed shearing; AOs-P: AOs fabricated by high-pressure homogenization; AOs-U: AOs fabricated by ultrasonication. Results with different letters within the same pattern are significantly different (p < 0.05).

As illustrated in Fig. 4, all AOs demonstrated significant free radical scavenging capabilities, predominantly due to the incorporation of rose essential oil. Eugenol, a principal constituent of rose essential oil, plays a pivotal role in its antioxidant efficacy [49]. Furthermore, at equivalent concentrations, the DPPH radical scavenging rate of AOs surpassed that of ABTS radicals, a phenomenon likely attributable to the hydrophobic characteristics of the essential oil, which augment its antioxidant activity in alcoholic environments. This observation is consistent with our prior research, where oregano essential oil emulsions also exhibited a higher scavenging efficiency for DPPH radicals compared to ABTS radicals [50]. The method of emulsification was also found to exert a substantial impact on the antioxidant properties of the AOs. Notably, AOs synthesized via ultrasonication exhibited enhanced antioxidant activity relative to those produced through high-pressure homogenization and high-speed shearing. This enhancement is presumably linked to the reduced particle size of the ultrasonically prepared AOs (as shown in Fig. 2 A). Previous investigation into AOs have similarly documented that smaller particle sizes correlate with more robust free radical scavenging abilities [21]. Researchers have ascribed this enhanced activity to the diminutive particle size, which promotes superior dispersion within the system and increases the interfacial area between antioxidant constituents and free radicals, thereby augmenting the overall antioxidant efficacy.

### 3.8 Light transmittance of AOs coatings

**Fig. 5:**
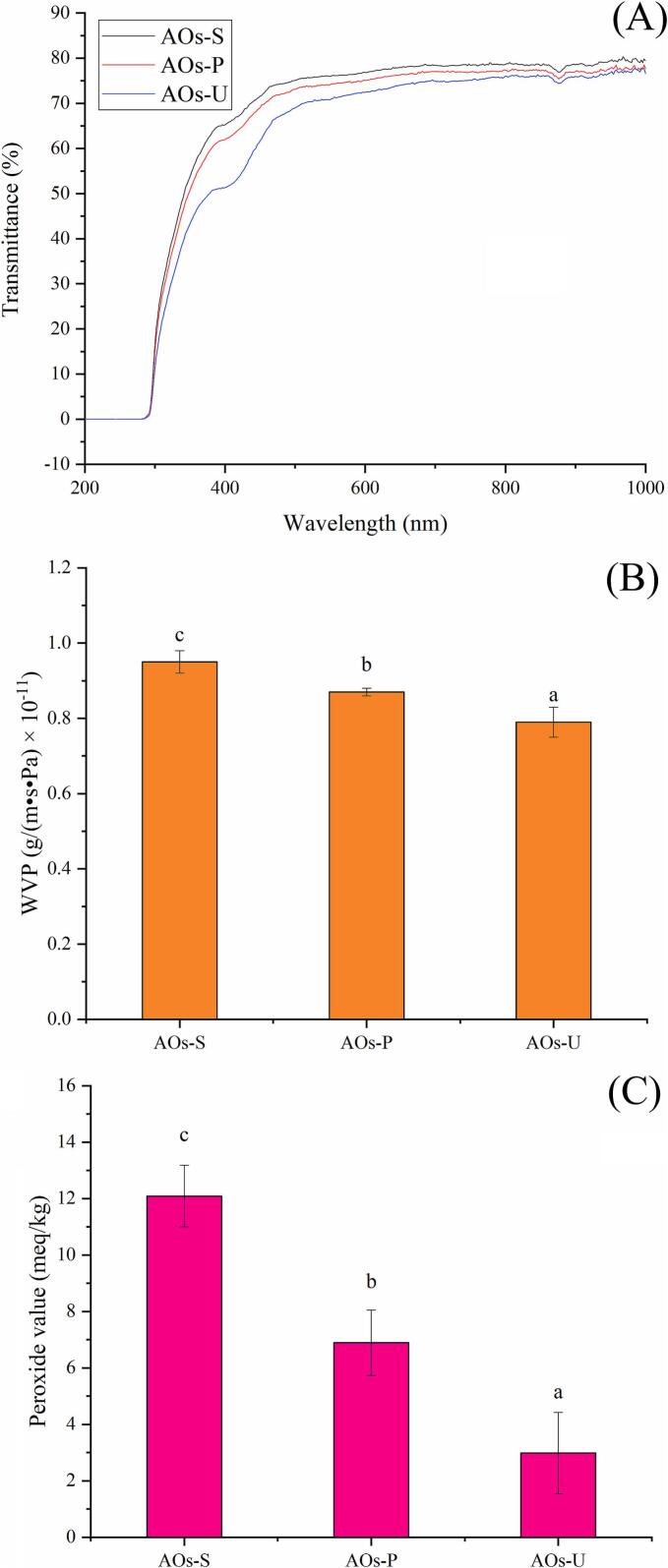
Transparency (A), water vapor permeability (B) and peroxide value (C) of artificial oleosomes (AOs) stabilized by hemp seed oil body proteins (OBPs). AOs-S: AOs fabricated by high-speed shearing; AOs-P: AOs fabricated by high-pressure homogenization; AOs-U: AOs fabricated by ultrasonication. Results with different letters within the same pattern are significantly different (p < 0.05).

As demonstrated in Fig. 5 A, the emulsification method exerted a significant influence on the light transmittance of coatings formed by AOs. Specifically, AOs prepared via ultrasonication exhibited markedly lower light transmittance across the wavelength range of 300–1000 nm compared to those fabricated using high-pressure homogenization and high-speed shearing. This phenomenon can be attributed to the cavitation effect induced by ultrasonication, which generates smaller particle sizes in the AOs (as shown in Fig. 2 A). The reduction in particle size facilitates a more uniform dispersion of the essential oil within the coatings, thereby enhancing light obstruction. This finding aligns with the earlier discussion on the microscopic structure of AOs, where ultrasonically prepared AOs displayed a more homogeneous distribution with reduced inter-particle spacing. In contrast, AOs produced by high-speed shearing exhibited an irregular distribution characterized by larger gaps between individual AOs. These results are consistent with prior studies on essential oil emulsion films, which similarly reported that emulsions with smaller particle sizes promote a more uniform distribution of the essential oil within the film matrix, consequently lowering the film’s light transmittance [16,17].

### 3.9 Water vapor permeability and oxygen barrier property of AOs coatings

As illustrated in Fig. 5 B and C, the emulsification method exerted a significant influence on the water vapor and oxygen barrier properties of coatings derived from AOs. Notably, AOs prepared via ultrasonication demonstrated superior barrier performance against both water vapor and oxygen compared to those fabricated using high-pressure homogenization and high-speed shearing. This enhancement can be attributed to the smaller particle size and more homogeneous distribution of ultrasonically prepared AOs (as shown in Fig. 2 A), which minimize interparticle voids and thereby hinder the permeation of water vapor and oxygen. These findings align with previous studies on emulsion-based films of essential oil, which similarly reported that emulsions with reduced particle sizes yield films with enhanced barrier properties [16,17]. Researchers have ascribed this improvement to the increased surface hydrophobicity and denser microstructure of films formed by smaller particles. In light of these findings, the influence of emulsification techniques on the surface hydrophobicity and microstructural characteristics of AOs coatings necessitates a more comprehensive exploration, as elaborated in the following sections of this study.

### 3.10 Contact angle of AOs coatings

**Fig. 6:**
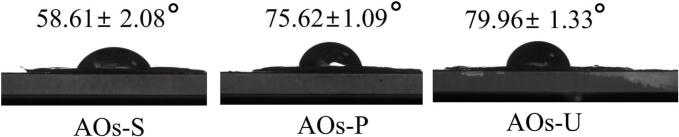
Contact angle of coatings produced by artificial oleosomes (AOs). AOs-S: AOs coatings fabricated by high-speed shearing; AOs-P: AOs coatings fabricated by high-pressure homogenization; AOs-U: AOs coatings fabricated by ultrasonication.

The contact angle is widely utilized as a key parameter to evaluate the surface hydrophobicity of films or coatings, which critically influences their wettability and moisture transmission characteristics. Theoretically, the contact angle ranges from 0° to 180°, representing the extremes of complete liquid spreading (perfect wetting) and absolute non-wetting, respectively [51]. A higher contact angle corresponds to a more hydrophobic surface, whereas a lower angle indicates hydrophilicity. For emulsion-based edible films intended for packaging or coating applications, high surface hydrophobicity is essential, necessitating contact angles as large as possible. A quantitative threshold for hydrophobicity has been established, with surfaces exhibiting water contact angles exceeding 65° classified as hydrophobic [52]. As depicted in Fig. 6, the emulsification method exerted a significant impact on the surface hydrophobicity of AOs coatings. Notably, only AOs coatings prepared via high-pressure homogenization and ultrasonication demonstrated contact angles above 65°, indicating that these methods yield AOs coatings meeting the criteria for hydrophobic materials. Furthermore, the contact angle results were consistent with the observed water vapor barrier performance (as shown in Fig. 5 B), suggesting that enhanced surface hydrophobicity contributes to the superior water vapor barrier properties of ultrasonically prepared AOs coatings. This finding aligns with prior study on emulsion-based films, which similarly reported that increased surface hydrophobicity correlates with improved water vapor barrier performance [53].

### 3.11 Intermolecular interactions of AOs coatings

**Fig. 7:**
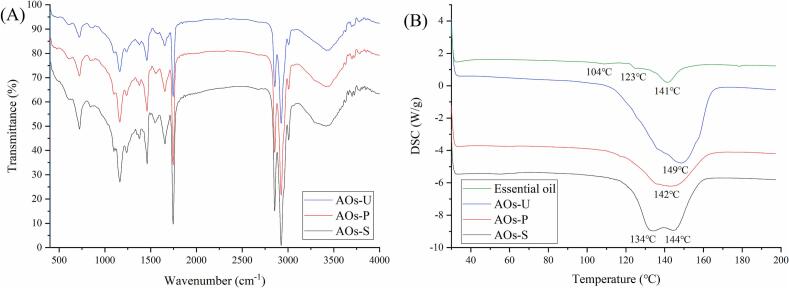
Fourier transform infrared spectroscopy spectra (A) and differential scanning calorimetry curves (B) of coatings produced by artificial oleosomes (AOs). AOs-S: AOs coatings fabricated by high-speed shearing; AOs-P: AOs coatings fabricated by high-pressure homogenization; AOs-U: AOs coatings fabricated by ultrasonication.

As depicted in Fig. 7 A, the infrared spectrum revealed an absorption peak at 3200 cm^−1^, corresponding to the O-H stretching vibration, which is commonly employed to investigate intermolecular hydrogen bonding interactions within the films or coatings [53]. The emulsification method exerted a significant influence on the absorption intensity at 3200 cm^−1^. Notably, the AOs coatings prepared via ultrasonication exhibited a markedly higher transparence compared to those fabricated using high-pressure homogenization and high-speed shearing. This observation suggests weaker hydrogen bonding interactions in ultrasonically prepared AOs, likely attributable to their elevated net charge (as shown in Fig. 2 B), which impedes the formation of hydrogen bonds. The diminished hydrogen bonding interactions further account for the lower apparent viscosity (as shown in Fig. 2 D) observed in ultrasonically prepared AOs. In the infrared spectrum, the three peaks around at 2900 cm^−1^ are indicative of fatty acid vibrations [54]. The ultrasonically prepared AOs coatings also demonstrated a higher transparence, implying effective encapsulation of the essential oil within the AOs. Similarly, at 1700 cm^−1^, which corresponds to the vibration of free fatty acids [54], the ultrasonically prepared AOs coatings exhibited a higher transparence. This further corroborates the efficacy of the ultrasonication method in encapsulating the essential oil, highlighting its potential for enhancing the stability and functionality of AOs coatings.

### 3.12 Thermal property of AOs coatings

As illustrated in Fig. 7 B, the endothermic peak observed within the temperature range of 100 °C to 160 °C is likely attributable to the volatilization of essential oil. This finding is consistent with prior research on essential oil films and microcapsules, which has similarly identified the volatilization temperature of essential oils to range between 100 °C and 250 °C [16,55]. For the free essential oil, multiple endothermic peaks were observed starting from 100 °C, which may be attributed to the sequential evaporation of different volatile components. In the AOs coatings fabricated via high-speed shearing, two distinct endothermic peaks were prominently identified. The initial peak is presumed to correspond to the volatilization of free essential oil, whereas the subsequent peak likely signifies the volatilization of essential oil encapsulated within the AOs. Conversely, AOs coatings produced through high-pressure homogenization and ultrasonication exhibited only a single endothermic peak. This observation is in agreement with the infrared spectroscopy results (as shown in Fig. 7 A), confirming that AOs coatings prepared by high-pressure homogenization and ultrasonication effectively encapsulate the essential oil. Moreover, the volatilization temperature of essential oil in the ultrasonically prepared AOs coatings was higher than that in the coatings prepared by high-pressure homogenization, indicating that the ultrasonically prepared AOs coatings possess enhanced thermal stability. Previous investigation on essential oil microcapsules have consistently demonstrated a positive correlation between encapsulation efficiency and the temperature required for essential oil volatilization, with higher encapsulation rates resulting in elevated volatilization temperatures [55]. Furthermore, the AOs prepared by ultrasonication exhibited significantly larger endothermic peak areas compared to those prepared by the other two methods, indicating that more heat absorption was required for the phase transition of essential oils in these AOs. This phenomenon may be attributed to their more stable structure, further demonstrating their superior thermal stability.

### 3.13 Surface morphology and topography of AOs coatings

**Fig. 8:**
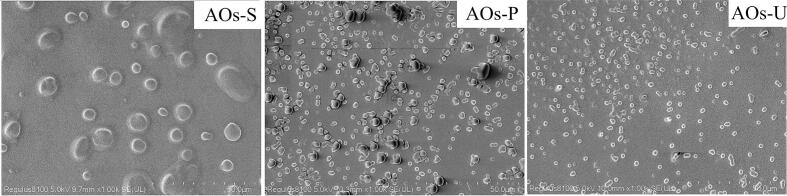
Scanning electron microscope images of the coatings produced by artificial oleosomes (AOs). AOs-S: AOs coatings fabricated by high-speed shearing; AOs-P: AOs coatings fabricated by high-pressure homogenization; AOs-U: AOs coatings fabricated by ultrasonication.

**Fig. 9:**
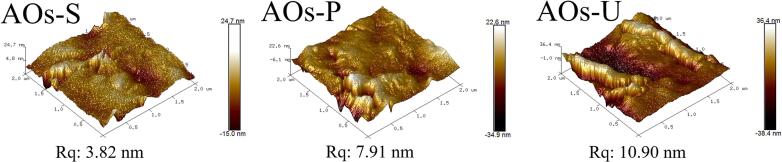
Atomic force microscope images of the coatings produced by artificial oleosomes (AOs). AOs-S: AOs coatings fabricated by high-speed shearing; AOs-P: AOs coatings fabricated by high-pressure homogenization; AOs-U: AOs coatings fabricated by ultrasonication.

As illustrated in Fig. 8, the AOs coatings fabricated using distinct emulsification techniques exhibit markedly different surface morphologies. The coatings prepared via high-speed shearing are characterized by the predominance of large aggregates with significant inter-particle voids, a structural feature that elucidates their higher light transmittance and reduced gas barrier performance (as shown in Fig. 5). In contrast, the coatings produced through high-pressure homogenization and ultrasonication are primarily composed of smaller aggregates, with the ultrasonically prepared coatings displaying a more homogeneous distribution of AOs across the surface. Furthermore, the presence of these AOs aggregates contributes to an increase in surface roughness. To quantify this, surface roughness was analyzed using AFM. As demonstrated in Fig. 9, the coatings with predominantly small and uniformly distributed aggregates exhibit greater surface roughness, as indicated by the higher Rq value observed in the ultrasonically prepared coatings. This enhanced roughness is directly associated with the improved surface hydrophobicity of the ultrasonically prepared AOs coatings (as shown in Fig. 6). These findings are consistent with prior research on edible films, which has established a positive correlation between increased surface roughness and enhanced hydrophobicity [15].

### 3.14 Preservation effect of AOs coatings on bananas

**Fig. 10:**
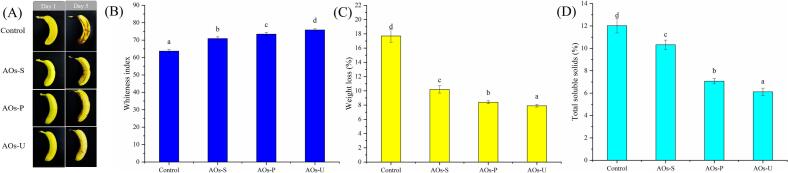
Changes in appearance (A), whiteness index (B), weight loss (C) and total soluble solids (D) of bananas after AOs coatings. AOs-S: AOs coatings fabricated by high-speed shearing; AOs-P: AOs coatings fabricated by high-pressure homogenization; AOs-U: AOs coatings fabricated by ultrasonication. Results with different letters within the same pattern are significantly different (p < 0.05).

Bananas are a perishable fruit with a typically short shelf life, and their ripening process can be directly monitored through visual changes. Post-harvest, as the storage period extends, browning on the banana surface occurs due to the formation of quinones from polyphenols catalyzed by polyphenol oxidase [56]. As depicted in Fig. 10 A, bananas without AOs coatings exhibit noticeable browning after 5 days of storage. In contrast, bananas treated with AOs coatings, especially those prepared by ultrasound, show minimal browning. This can be attributed to the high oxygen barrier property of the ultrasound-prepared AOs coatings (as shown in Fig. 5 C), effectively inhibiting enzymatic browning. Fig. 10 B illustrates the impact of emulsification technology on the whiteness of bananas during storage, consistent with visual observations, showing that bananas treated with ultrasound-prepared AOs coatings have the highest whiteness values. Furthermore, ultrasound-prepared AOs coatings exhibit the best water retention, as evidenced by their lower weight loss (Fig. 10 C). This can also be ascribed to the high moisture barrier property (as shown in Fig. 5 B) and surface hydrophobicity (as shown in Fig. 6) of the ultrasound-prepared AOs coatings. In addition, changes in soluble solids content during storage are crucial indicators of banana freshness. Post-harvest, carbohydrates in bananas gradually convert to soluble solids during storage [57]. AOs coatings treatment can form a semi-permeable film, reducing the respiration rate of bananas [27]. As expected, Fig. 10 D shows that all AOs coatings-treated bananas have lower soluble solids content, with bananas treated with ultrasound-prepared AOs coatings exhibiting the slowest ripening rate. This might be related to their superior gas barrier properties (as shown in Fig. 5).

## 4 Conclusion

This work mechanistically decouples the relationship between emulsification methods and AOs functionality. Ultrasonication-induced cavitation facilitated OBPs structural rearrangement, exposing hydrophobic domains and enhancing electrostatic stabilization. These interfacial modifications enabled the formation of compact, thermally stable coatings with hierarchical microstructures that synergistically impede gas diffusion and light penetration. The delayed banana ripening underscores the translational potential of ultrasonicated AOs in perishable food protection. Future research directions should focus on: OBPs-essential oil molecular docking mechanisms, in vivo bioavailability of encapsulated actives, and life-cycle assessment of AOs production scalability. This technology platform opens avenues for next-generation bio-based preservatives addressing global food waste challenges.

## CRediT authorship contribution statement

**Haoran Zhu:** Writing – original draft. **Yuhan Cao:** Data curation. **Xinyu Zhang:** Investigation. **Qin Hu:** Data curation. **Feng Xue:** Writing – review & editing, Funding acquisition.

## Declaration of competing interest

The authors declare that they have no known competing financial interests or personal relationships that could have appeared to influence the work reported in this paper.
